# Insight into the Binding Interaction between PEDCs and hERRγ Utilizing Molecular Docking and Molecular Dynamics Simulations

**DOI:** 10.3390/molecules29143256

**Published:** 2024-07-10

**Authors:** Fanqiang Bu, Lin Chen, Ying Sun, Bing Zhao, Ruige Wang

**Affiliations:** 1 College of Chemistry and Chemical Engineering, Qiqihar University, Qiqihar 161006, China; fanqiangbu@yeah.net (F.B.); m13895721559@163.com (Y.S.); 18304521342@163.com (B.Z.); 2 State Key Laboratory of Organic-Inorganic Composites, Beijing Laboratory of Biomedical Materials, Beijing University of Chemical Technology, Beijing 100029, China; 3 Heilongjiang Provincial Key Laboratory of Surface Active Agent and Auxiliary, Qiqihar University, Qiqihar 161006, China

**Keywords:** hERRγ, PEDC, molecular docking, MD simulation, MM-PBSA, SIE

## Abstract

Phenolic environmental endocrine-disrupting chemicals (PEDCs) are persistent EDCs that are widely found in food packaging materials and environmental media and seriously threaten human health and ecological security. Human estrogen-related receptor γ (hERRγ) has been proposed as a mediator for the low-dose effects of many environmental PEDCs; however, the atomic-level descriptions of dynamical structural features and interactions of hERRγ and PEDCs are still unclarified. Herein, how three PEDCs, 4-(1-methylpropyl)phenol (4-sec-butylphenol), 5,6,7,8-tetrahydro-2-naphthol (tetrahydro-2-napthol), and 2,2-bis(4-hydroxy-3,5-dimethoxyphenyl)propane (BP(2,2)(Me)), interact with hERRγ to produce its estrogenic disruption effects was studied. Molecular docking and multiple molecular dynamics (MD) simulations were first conducted to distinguish the detailed interaction pattern of hERRγ with PEDCs. These binding structures revealed that residues around Leu271, Leu309, Leu345, and Phe435 are important when binding with PEDCs. Furthermore, the binding energies of PEDCs with hERRγ were also characterized using the molecular mechanics/Poisson Boltzmann surface area (MM-PBSA) and solvated interaction energy (SIE) methods, and the results showed that the interactions of CH-π, π-π, and hydrogen bonds are the major contributors for hERRγ binding to these three PEDCs. What is striking is that the methoxide groups of BP(2,2)(Me), as hydrophobic groups, can help to reduce the binding energy of PEDCs binding with hERRγ. These results provide important guidance for further understanding the influence of PEDCs on human health problems.

## 1. Introduction

Environmental endocrine-disrupting chemicals (EDCs), which are widely found in food packaging materials and environmental media, can affect the synthesis, release, transmission, binding, and so on of hormones, resulting in biological developmental, reproductive, immune, neurological, or metabolic diseases in organisms [1,2,3]. Among them, phenolic EDCs (PEDCs) are toxic organic contaminants [4]. They have negative effects on the reproductive system, nervous system, and immune system of organisms, mainly intruding into the human body through the alimentary tract, respiratory system, and skin, binding to proteins in the cell protoplasm, and causing cell inactivation; they may even cause spinal cord stimulation, leading to systemic poisoning [5,6]. Therefore, many efforts have been made to investigate the effects of PEDCs on mammalian systems [7,8,9].

PEDCs can cause various health problems in organisms by affecting the normal functions of the reproductive, neural, and immune systems [10]. Estrogen-related receptors (ERRs), a nuclear receptor transcription factor family [11], have three members, namely ERRα, ERRβ, and ERRγ, which act in various biologic processes such as cell energy metabolism [12]. ERRs mainly consist of an N-terminal transactivation domain, a central DNA-binding domain, and a C-terminal ligand-binding domain (LBD), as shown in Figure S1 [13]. The LBD consists of two antiparallel β-sheets (hereafter referred to as S1 and S2) and twelve α-helices (consisting of H1 to H12), and its hydrophobic binding pockets are composed of S1, S2, H3, H6, H7, H8, and H11. Human ERRγ (hERRγ), a constitutive active nuclear receptor, serves as a mediator for many low-dose effects of PEDCs. Through structural analysis, it was found that the majority of PEDCs in the external environment can bind in the pockets of hERRγ, thereby interfering with downstream signaling pathways [14]. Meanwhile, as hERRγ manages the expression of various disease-related genes, for example, those involved in cancer and adiposity, when its normal function is affected by exogenous compounds, the normal functioning of organisms could be severely affected [15,16,17]. Numerous studies have demonstrated that PEDCs can disrupt the normal functioning of nuclear receptors and also regulate the basic activity of hERRγ. Among these PEDCs, 4-sec-butylphenol and tetrahydro-2-napthol are important raw materials in many industrial products and are potential threats to people’s health. Furthermore, BP(2,2)(Me), a sustainable bisphenol from lignin, is considered a promising alternative for commercial bisphenol [18]. A previous study by Thouennon et al. showed that the capacity of 4-sec-butylphenol and tetrahydro-2-napthol to bind with hERRγ is low, with EC_50_ values of approximately 109 and 260 nM, respectively [14]. Furthermore, Amitrano et al. revealed through molecular docking methods that the ability of BP(2,2)(Me) to bind with ERα is weak [18]. However, the atomic-level descriptions of the dynamical structural features and interactions of PEDCs with hERRγ are still unclarified. Therefore, exploring the mode of interaction between PEDCs and hERRγ is essential to uncovering the endocrine-disrupting mechanisms of PEDCs, which can help us to fully assess the latent diseases related to PEDC exposure.

**Figure 1:**
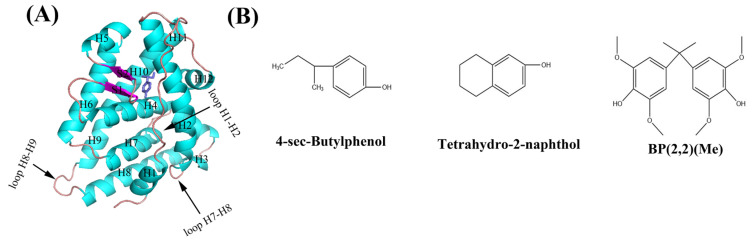
Structures of the complex of hERRγ with 4-sec-butylphenol ((**A**), PDB ID: 6I66) and the three PEDCs (**B**) in our study.

Molecular docking is a popular approach used to predict the interactions between small ligands and the appropriate targets [19]. While there are a variety of docking programs in use, new computational methods based on artificial intelligence and machine learning are expected to make an even greater contribution [20,21,22,23]. In the past few decades, receptor–ligand interactions were broadly analyzed using traditional molecular dynamics (MD) simulations [24,25,26,27]. However, the conformations sampled by this approach may be swallowed in the locally minimal space, thereby resulting in under-sampling. As a valuable method, multiple MD simulations may preferably obtain the conformation of receptor–ligand interactions and can describe the dynamic and detailed interactions of receptors and ligands [28,29,30,31,32,33], which is a difficult task for experimental studies. Hence, in our work, the complexes of hERRγ with 4-sec-butylphenol, tetrahydro-2-napthol, and BP(2,2)(Me) (Figure 1) were taken as the research targets, and we explored the interaction mechanism between PEDCs and hERRγ using molecular docking, multiple MD simulations, and the MM-PBSA and SIE methods [34,35]. The dynamical structural features and interactions between hERRγ and PEDC were investigated, and the crucial residue distribution was assessed in our study. These results may offer new perspectives on the estrogen-disrupting effects of PEDCs.

## 2. Results and Discussion

### 2.1. Dynamics of hERRγ-PEDC Complexes

To assess the stability of MD simulations of hERRγ-4-sec-butylphenol, hERRγ-tetrahydro-2-napthol, and hERRγ-BP(2,2)(Me) complexes, the root-mean-square deviations (RMSDs) for the hERRγ backbone atoms in MD simulations were evaluated compared with the corresponding original structure (Figure S2). After approximately 50 ns of simulations, the RMSD values in each hERRγ complex showed high stability. Therefore, the last 200 ns equilibrated portions of three replicas were connected, and each system possessed 600 ns trajectories for subsequent data analysis. The mean RMSD values of the hERRγ and 4-sec-butylphenol, tetrahydro-2-napthol, and BP(2,2)(Me) complexes were 2.31, 2.20, and 2.17 Å, respectively, indicating that the conformation of hERRγ in these complexes did not exhibit major structural change during the simulations. The experimental results of Thouennon et al. [14] also demonstrated the stability of hERRγ, as seen in the change in the hERRγ structure over time in Figure S3. In addition, to further evaluate the stability of the MD simulations, the enthalpy and entropy values were calculated (Figures S4 and S5). There were slight fluctuations in the calculated enthalpy and entropy between the different structures selected, but their cumulative average enthalpy and entropy values quickly stabilized. From the above analysis, it can be judged that the three complexes were stable in the 600 ns simulations. Therefore, post-processing analysis focused on these stable MD trajectories.

To investigate the detailed residual fluctuations in hERRγ interacting with different PEDCs, the root-mean-square fluctuations (RMSFs) in hERRγ Cα in the simulations related to the average coordinates of the joined 600 ns trajectory were computed. As shown in Figure S6, the flexibility patterns of residues in hERRγ were similar in these complexes. Among these complexes, residues near the active pockets composed of H2, H3, H5, H6, H10, S1, and S2 displayed a high degree of stability. This is mainly because the catalytic function of hERRγ requires a stable three-dimensional structure. However, the residues at both ends of hERRγ and around its loops showed high flexibility. It is worth noting that the residues around loops H1–H2, S1–S2, H7–H9, and H11–H12 exhibited distinct flexibility. In particular, loops such as S1–S2, H7–H8, and H10–H11 interacting with 4-sec-butylphenol and loop H1–H2 interacting with tetrahydro-2-naphthol showed high flexibility. The influence of environmental PEDCs on the internal dynamics of hERRγ was studied. This was performed using the ascertained correlated fluctuations in Cα (Figure S7). Obviously, in these complexes, the correlated motions of the D1 region, composed of H2 and H3–H4; the D2 region, composed of H2, s1, s2, and H5; the D3 region, composed of H7–H8 and H3–H4; the D4 region, composed of H5–H7 and H4–H5; the D5 region, composed of H10–H12 and H5–H6; and the D6 region, composed of H7–H8 and H8–H9, are more apparent. When hERRγ interacts with different PEDCs, the differences in its internal dynamics are the manifestation of changes in the relative position of residues in these complexes. The result is similar to the results of the RMSF analysis. In summary, these structural changes may result in changes in the binding affinity between hERRγ and PEDCs.

### 2.2. Analysis of Free Energy between hERRγ and PEDCs

To gain insight into the source of energy differences in hERRγ with different PEDCs, the contributions of different energy terms were ascertained using the MM-PBSA approach (Table 1). Obviously, the calculated energies between hERRγ and 4-sec-butylphenol and between hERRγ and tetrahydro-2-napthol were −5.94 and −5.67 kcal/mol, respectively. These calculated energies have a good correlation with the experimental values for the hERRγ-4-sec-butylphenol (−9.49 kcal/mol) and hERRγ-tetrahydro-2-napthol (−8.98 kcal/mol) complexes [14]. When errors are taken into account, the energy results obtained are of the same order. In addition, the calculated energy of the hERRγ-BP(2,2)(Me) complex was −2.97 kcal/mol, which proves that BP(2,2)(Me) has weak interference ability on hERRγ; this is similar to the effect of BP(2,2)(Me) on ERα [18]. Nevertheless, the MM-PBSA method could not precisely reproduce the results from the experiment. The main factors include the following: (a) an explicit solvent was used in the MD simulation, while an implicit solvent was used in the MM-PBSA calculation [36]; (b) the intermediate electric constant (e) changes in the MD simulation, while the internal (e) is constant in the calculation with the MM-PBSA method [37]; and (c) there is less conformational entropy sampling in the MM-PBSA calculation [38,39]. In our work, we mainly performed the MM-PBSA method to calculate the discrepancies in the binding of PEDCs to hERRγ, rather than to reproduce the experimental results. Thus, the method is very suitable for calculating the energy of different EDCs for hERRγ. By comparing the separate energy components of these various complexes in Table 1, we observed that the chief driving force of hERRγ interacting with different PEDCs is van der Waals energy (ΔE_vdW_) from nonpolar energy. The ΔE_vdW_ values between hERRγ and 4-sec-butylphenol, tetrahydro-2-napthol, and BP(2,2)(Me) were −23.95, −23.54, and −46.47 kcal/mol, respectively. In addition, in these complexes, electrostatic interaction energy (ΔE_ele_) makes positive contributions to the binding of hERRγ with different PEDCs. The result conforms with the subsequent analysis of hydrogen bonds. However, polar solvation interactions destroy the interactions between different PEDCs and hERRγ, while nonpolar solvation interactions help them to interact. Moreover, the entropy (−TΔS) values of 4-sec-butylphenol, tetrahydro-2-napthol, and BP(2,2)(Me) with hERRγ were 15.21, 15.66, and 20.52 kcal/mol, respectively. These differences are mainly due to the vibrational entropy caused by structural changes when hERRγ interacts with different PEDCs.

To confirm the calculation results from the MM-PBSA method, the energies of hERRγ interacting with different PEDCs were also assessed based on the SIE approach (Table 2). The free energies in hERRγ with 4-sec-butylphenol, tetrahydro-2-napthol, and BP(2,2)(Me) were −6.68, −6.54, and −5.68 kcal/mol, respectively. The results calculated using the SIE method agree well with the results from experiments [14,18]. The results of the above two energy calculations show that they can be used to study the interaction pattern of different PEDCs with hERRγ. As displayed in Table 2, in these PEDC and hERRγ complexes, the key interactions are the nonpolar interactions (ΔG_nonpol_), including the ΔE_vdW_ and nonpolar contributions (ΔG_cav_). Thus, the primary driving forces of the interactions between these PEDCs and hERRγ are hydrophobic interactions. Although the intermolecular Coulomb interactions (ΔE_Coul_) are important for their binding, this effect is offset by the reaction energies (ΔG^R^), which are between 4.17 and 9.96 kcal/mol. These energies (ΔG^R^) are not conductive to the interaction of different PEDCs with hERRγ. ΔE_Coul_ also has a positive effect on PEDCs interacting with hERRγ. In light of the above MM-PBSA and SIE energy analyses, it can be seen that hydrophobic interactions composed of van der Waals energies are involved in the stability of PEDC and hERRγ complexes, which is also consistent with the results from the experiments [14,18].

### 2.3. Structure and Energy Relationship between hERRγ and PEDCs

**Figure 2:**
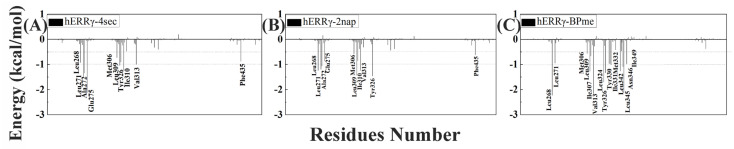
Contributions of key residues when interacting with different PEDCs: (**A**) 4-sec-butylphenol; (**B**) tetrahydro-2-napthol; (**C**) BP(2,2) (Me).

**Figure 3:**
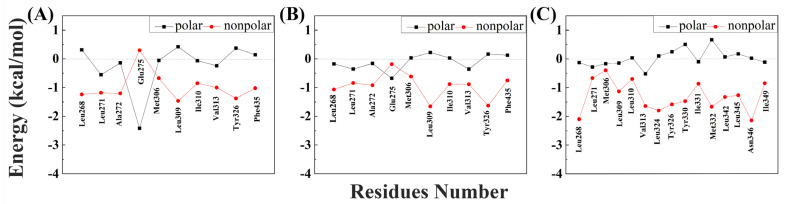
Nonpolar interaction and polar interaction energy of important residues when interacting with different PEDCs: (**A**) 4-sec-butylphenol; (**B**) tetrahydro-2-napthol; (**C**) BP(2,2)(Me).

**Figure 4:**
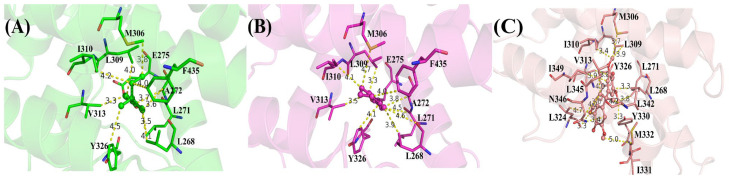
Interactions of different key residues when interacting with different PEDCs: (**A**) 4-sec-butylphenol (blue); (**B**) tetrahydro-2-napthol (magentas); (**C**) BP(2,2)(Me) (oranges).

To better understand the binding pattern of PEDCs and hERRγ, the hotspot residues of hERRγ when interacting with PEDCs were analyzed (Figure 2). Generally, the sources of important contributions to their interactions come from four regions around Leu271, Leu309, Leu345, and Phe435. It can be seen that the hydrophobic residues are important residues of the different PEDCs interacting with hERRγ, for example leucine. The experimental results almost agree with the dynamic structure analysis [14]. For example, residues Glu275 and Arg316 form hydrogen bonds with 4-sec-butylphenol. Phe435 also forms C-H/π interactions with 4-sec-butylphenol. Almost all of the important residues that interact with different PEDCs have weak polar interactions and strong nonpolar interactions, as shown in Tables S1–S3 and in Figure 3. Among the residues that interact with different PEDCs, except for residue Glu275, many of those involved in polar interactions form hydrogen bonds, as shown in Figure 4 and Table S4; those with small probabilities are not listed.

For the hERRγ-4-sec-butylphenol complex, there were 10 residues with energies that contribute more than −0.5 kcal/mol (Figure 2A). Among these, the residues Leu271, Ala272, Glu275, Leu309, Val313, and Tyr326 provide over −1 kcal/mol of energy, which is important for interacting with 4-sec-butylphenol. Based on the nonpolar interactions (Figure 3A), the absolute values for the residues Leu268, Leu271, Ala272, Leu309, and Tyr326 exceeded −1 kcal/mol (Table S1). In this study of polar interactions, the absolute value of only one residue, Glu275, was more than 1 kcal/mol (Figure 3A); the spatial distribution is shown in Figure 4A. The energies between 4-sec-butylphenol and Leu268, Leu271, and Val313 are −0.92, −1.49, and −1.24 kcal/mol, respectively. This is mainly due to the interactions between the hydrophobic alkyls of these residues and the benzene group of 4-sec-butylphenol. The alkyl group of residue Ala272 is situated around the benzene ring of 4-sec-butylphenol, forming CH-π interactions. Thus, Ala272 binding with 4-sec-butylphenol contributes an energy value of −1.34 kcal/mol. The interaction of CH-HC between the side chain of Met306 and the alkyl of 4-sec-butylphenol is favorable for the interaction between hERRγ and 4-sec-butylphenol (−0.73 kcal/mol). The hydrophobic benzene ring of 4-sec-butylphenol can contact the isobutyl–methyl ring of Leu309 and the benzene ring of Tyr326 to form CH-π and T-shaped π-π, respectively. The results showed that residues Leu309 and Tyr326 contributed energies of nearly −1.04 and −1.00 kcal/mol, respectively. The electrostatic solvation interaction of residue Tyr326 is 0.37 kcal/mol, which impedes the interaction of hERRγ and 4-sec-butylphenol. In addition, the alkyl of Ile310 and the alkyl of 4-sec-butylphenol produce an energy of −0.91 kcal/mol. The van der Waals energy (−0.89 kcal/mol) for the hydrophobic group of Phe435 with the hydrophobic side chain of 4-sec-butylphenol is one of the key causes for the interaction between hERRγ and 4-sec-butylphenol (Figure 4A). Furthermore, Glu275 supplies −2.12 kcal/mol energy with 4-sec-butylphenol, primarily since the hydrogen bond occupancy of Glu275-OE1-H⋯OAC-4-sec-butylphenol is approximately 86.93% (Table S4).

For the hERRγ-tetrahydro-2-napthol complex, the energy contributions of 10 residues exceed −0.5 kcal/mol (Figure 2B); Leu268, Leu271, Ala272, Leu309, Val313, and Tyr326 offer over −1 kcal/mol of energy. In our study of nonpolar interaction energies (Figure 3B), three residues, Leu268, Leu309, and Val313, possessed absolute values exceeding 1 kcal/mol (Table S2). The residues Met306, Ile310, and Val313, which closely resemble those for the hERRγ-4-sec-butylphenol complex, also contribute to a major interaction. The reason why these residues play a major role in hERRγ-tetrahydro-2-napthol is similar to that for the hERRγ-4-sec-butylphenol complex (Figure 4B). The difference is that the C-H⋯O interaction between the tetrahydro-2-napthol and carbonyl oxygen of Leu268 causes the backbone electrostatic energy of Leu268 to be −0.58 kcal/mol, so the total energy is increased by 0.32 kcal/mol. The alkyl of Leu271 is far away from the hydroxyl group of 4-sec-butylphenol, which is not conducive to the formation of CH-HO interactions. Therefore, the total contribution of Leu271 with tetrahydro-2-napthol is −1.19 kcal/mol. Compared with that in the hERRγ-4-sec-butylphenol complex, the energy of Ala272 with tetrahydro-2-napthol decreases by 0.27 kcal/mol. This results from the formation of a relatively weak interaction of the alkyl group of residue Ala272 with the hydrophobic benzene ring of tetrahydro-2-napthol. The interaction energy of Leu309 with tetrahydro-2-napthol increases by about 0.39 kcal/mol. This is connected with strengthened hydrophobic interactions of the alkyl ring of L309 with the benzene group of tetrahydro-2-napthol. Through comparison with the hERRγ-4-sec-butylphenol complex, we see that the binding pattern of tetrahydro-2-napthol with hERRγ resembles that of 4-sec-butylphenol with hERRγ, and the van der Waals energies (−1.47 and −0.63 kcal/mol) between the alkyls of residues Tyr326 and Phe435 and the hydrophobic benzene ring of tetrahydro-2-napthol are also a significant reason for their binding.

When hERRγ interacts with BP(2,2)(Me), there are 15 residues with energy over −0.5 kcal/mol (Figure 2C). Importantly, Leu268, Leu309, Val313, Leu324, Tyr326, Met332, Leu342, Leu345, and Asn346 make the largest contribution to BP(2,2)(Me), with amounts above −1 kcal/mol. As shown in this study of their nonpolar interaction energy (Figure 3C), the absolute values of the energy of nine residues, Leu268, Leu309, Val313, Leu324, Tyr326, Tyr330, Met33, Leu342, Leu345, and Asn346, are all over −1 kcal/mol (Table S3), and the spatial distribution of these important residues is depicted in Figure 4C. Nevertheless, nearly all of the primary residues binding to BP(2,2)(Me) have weak polar interactions. The energy of residue Leu268 in hERRγ-BP(2,2)(Me) bears a resemblance to that in the hERRγ-4-sec-butylphenol complex, playing an essential interaction role similar to that in hERRγ-4-sec-butylphenol. Leu309, Val313, Tyr326, Tyr330, and Ile331 have enhanced interactions with BP(2,2)(Me) compared with those in hERRγ-4-sec-butylphenol, which is related to enhanced interactions forming alkyl rings with the methoxy group of BP(2,2)(Me). Differently, the interactions of Leu271, Met306, and Ile310 with BP(2,2)(Me) are weakened. CO-H interactions were found for the carbonyl oxygen of Leu324 with the hydrophobic methoxy group of BP(2,2)(Me); therefore, electrostatic energy (−0.86 kcal/mol) is the primary interacting force. The interactions of Leu342 and Leu345 with BP(2,2)(Me) are also reinforced. They are associated with the enhanced interactions forming alkyl rings of these residues and alkyl groups of BP(2,2)(Me). Additionally, the hydrophobic alkyl group of Ile349 is located near the benzene ring of BP(2,2)(Me), forming a CH-π interaction. Thus, residue Ile349 offers approximately −0.97 kcal/mol to the interaction with BP(2,2)(Me).

On the whole, the binding of various PEDCs causes changes in their local structure by influencing the interactions with various residues. The most representative structural superimposition (Figures S8 and S9) demonstrates the significant change in loops H1-H2, H7-H8, and H8-H9. However, residues around Leu268, Val313, Leu345, and Phe435 can interact stably with various PEDCs, and CH-π, π-π, and hydrogen bond interactions between these PEDCs and important residues in hERRγ are the key to the stable existence of these complexes.

## 3. Materials and Methods

### 3.1. Molecular Docking

Two structures of hERRγ-4-sec-butylphenol and hERRγ-tetrahydro-2-napthol were obtained from RCSB (PDB ID: 6I66 and 6I67) [14]. Since there was no complex structure of hERRγ and BP(2,2)(Me), AutoDock 4.2 software [40] was used to dock BP(2,2)(Me) to the binding site of hERRγ. BP(2,2)(Me) was first optimized with the B3LYP method under the 6–31 g(d) basis set of the Gaussian09 package [41]. During docking, the coordinates of hERRγ were obtained by removing all crystal water and ligands based on the hERRγ-4-sec-butylphenol complex. The binding site of hERRγ with 4-sec-butylphenol was considered as the binding pocket for docking. Firstly, 4-sec-butylphenol was redocked with hERRγ to affirm the reliability of the docking method. After superimposing the experimental and docking structures, the conformation of the two structures was basically the same (Figure S10), indicating that the docking method was credible. Then, docking between BP(2,2)(Me) and hERRγ was performed using the graphical user interface of Auto Dock Tools. The semi-flexible docking method was performed to maintain the rigidity of hERRγ while allowing for the flexibility of the ligand. The grid box was set based on the binding pocket of the 4-sec-butylphenol ligand. The grid center coordinates of hERRγ in the X, Y, and Z directions were −15.03, −3.06, and −24.24, respectively. The size was set to 44 Å × 44 Å × 38 Å with a grid point spacing of 0.375 Å. The Lamarckian genetic algorithm was used for docking. One hundred optimal conformations were obtained for each hERRγ and PEDC docking result through the iterative search process. In the end, the complex with the lowest energy was retained for further analysis.

### 3.2. System Setup

Owing to the lack of parameters needed for the ligands 4-sec-butylphenol, tetrahydro-2-napthol, and BP(2,2)(Me) in the force field, optimization of these ligands was first conducted based on the Gaussian package. The missing hydrogen atoms were added to the corresponding heavy atoms of each system by applying the tLeap program in Amber 18 [42]. A standard amber (ff14SB) force field [43] was chosen to depict hERRγ, while a general amber force field [44] with AM1-BCC charges was employed to set the three PEDCs’ parameters. A truncated octahedral periodic box filled with TIP3P water [45] was used to process these systems. The distance forming the outermost atoms with the water box was over 12 Å. Generally, each system included more than 12,000 water molecules. In addition, to neutralize each simulated system, TIP3P water molecules were replaced with Na^+^.

### 3.3. MD Simulation

Prior to the MD simulations, to eliminate possible bad contact, each system was energetically minimized with complex atoms with force constants of 50 kcal mol^−1^ Å^−2^ through 4000 steepest descent steps and another 8000 conjugate gradient steps. Following that, canonical ensemble MD simulation was conducted for 600 ps, during which each system was heated progressively to 310 K at a constant force of 10 kcal mol^−1^ Å^−2^. Afterwards, an isothermal isobaric ensemble MD simulation for 1000 ps was applied with the complex atoms constrained. Ultimately, for each system under the *NPT* ensemble, three 250 ns simulations were performed. According to the Maxwell distribution, seeds with different values were randomly distributed to each repeated system. Long-range electrostatic interactions were calculated using the particle mesh Ewald method [46] with default values. The SHAKE algorithm [47] was constrained to bonds communicating with hydrogen atoms. The time step was 2 fs. Unless otherwise specified, the data in the figures and tables were analyzed based on connected 600 ns trajectories. PyMOL2.0 soft [48] was mainly conducted to imagine the trajectory and to portray structures. The MD simulations, including the energy minimization, were conducted using Amber18 soft with GPU-accelerated PMEMD [42].

### 3.4. MM-PBSA Calculation

The MM-PBSA method [49,50,51,52,53] implemented in amber18 was executed to calculate the binding free energy (ΔG_bind_) for the hERRγ-4-sec-butylphenol, hERRγ-tetrahydro-2-napthol, and hERRγ-BP(2,2)(Me) complexes. The energy was the values computed for 3000 structure samplings based on the equilibrated portions at 200 ps intervals. The calculation formulas are presented as follows:

(1) $$\Delta G_{bind}=G_{complex}-G_{\mathrm{receptor}}-G_{\mathrm{ligand}}$$

(2) $$\Delta G_{\mathrm{bind}}=\Delta H-T\Delta S$$

(3) $$\Delta H=\Delta E_{\mathrm{ele}}+\Delta E_{vdW}+\Delta G_{PB}+\Delta G_{SA}$$

(4) $$\Delta G_{\mathrm{SA}}=\gamma \Delta \mathrm{SASA}+\beta$$

ΔE_ele_, ΔE_vdW_, ΔG_PB_, and ΔG_SA_ represent the energies of electrostatic interaction, van der Waals interaction, polar solvation, and nonpolar solvation, respectively. ΔG_PB_ and ΔG_SA_ were estimated using the PB model and ΔSASA, respectively. The two empirical constants *γ* and *β* were 0.00542 kcal mol^−1^ Å^−2^ and 0.92 kcal mol^−1^, respectively. The ionic strength was 0.1 M, and the dielectric constants were 1.0 and 80.0 for the solute and surrounding solvent, respectively [54]. The entropy (TΔ*S*) was calculated via Normal-mode analysis [55] with the nmode module. Since the entropy calculation was expensive, only 100 structures based on 3000 structures were selected for calculation.

### 3.5. SIE Calculation

The SIE method [34,35,56] was performed to estimate the energy for the hERRγ-4-sec-butylphenol, hERRγ-tetrahydro-2-napthol, and hERRγ-BP(2,2)(Me) complexes. The calculation formulas are as follows:

(5) $$\Delta G_{bind}(\rho ,D_{in},\alpha ,\gamma ,C)=\alpha [E_{Coul}(D_{in})+E_{vdw}+\Delta G_{R}(\rho ,D_{in})+G_{cav}(\rho )+C]$$

(6) $$G_{\mathrm{cav}}=\gamma \Delta M_{SA}$$

E_Coul_, E_vdW_, ΔG^R^, and G_cav_ describe the intermolecular Coulomb, van der Waals, reaction, and nonpolar solvation energy, respectively. ΔG^R^ and G_cav_ were estimated using the boundary element method and ΔM_SA_, respectively.

The three empirical constants *ρ*, D_in_, and α represent the linear scaling factor of van der Waals radii and the internal dielectric invariable of the solute, respectively. The values of *ρ*, D_in_, α, γ, and C were 1.1, 2.25, 0.1048, 0.0129 kcal mol^−1^ Å^−2^, and −2.89 kcal mol^−1^, respectively. When calculating the energy using the Sietraj program for SIE calculations [34], the same 3000 structures as in the MM-PBSA calculation were chosen.

### 3.6. Conformational Dynamics Analysis

Based on the uniform sampling of equilibrium trajectories, the cross-correlation matrix [57] was calculated. The fluctuation of Cα atoms compared with their average positions in hERRγ during the simulation was used to assess the cross-correlation coefficient (*C_ij_*). The formula is as follows:

(7) $$C_{ij}=\frac{<\Delta r_{i}\Delta r_{j}>}{(<\Delta r_{i}^{2}><\Delta r_{j}^{2}>{)}^{2}}$$

The symbol Δ*r_i_* describes the displacement of the *i*-th atom from the mean position. The value of *C_ij_* is between −1 and 1. A positive *C_ij_* value shows positively correlated movement, while a negative *C_ij_* value shows negatively correlated movement.

### 3.7. Principal Component Analysis

To describe the collective motions of hERRγ during the simulation period, principal component analysis (PCA) [58,59,60] was conducted using ProDy2.0 software [61]. On the basis of uniform sampling of the equilibrium trajectory, the covariance matrix of the diagonal coordinate system was constructed through PCA. The movement analysis steps of hERRγ were, first, to project the trajectory in the direction described through the corresponding eigenvector and, second, to determine the main motion direction of hERRγ according to the first two eigenvalues. Through VMD 1.9.3 [62] software with the NMWiz plug-in, the 3D structure of hERRγ was visualized.

## 4. Conclusions

Molecular docking and MD simulations were executed to probe the conformational changes and interactions in hERRγ complexes with three PEDCs (4-sec-butylphenol, tetrahydro-2-napthol, and BP(2,2)(Me)). Furthermore, two energy computation methods (MM-PBSA and SIE) were used to uncover the important residues between them.

The docking and simulation results demonstrated that when these three PEDCs interact with hERRγ, residues near Leu268, Val313, Leu345, and Phe435 play significant roles in their interactions. Moreover, the energy analysis indicated that hydrophobic interactions resulting from van der Waals energies are involved in the stability of the binding between these three PEDCs and hERRγ, which is crucial in their interaction. In addition, the interactions of CH-π, π-π, and hydrogen bonds are major contributors to hERRγ binding to these three PEDCs. What is striking is that the hydrophobic methoxide groups of BP(2,2)(Me) can help to reduce the binding energy of PEDCs with hERRγ.

These findings clarify the details of these three PEDCs binding to hERRγ, which can provide estimable knowledge for further understanding of the influence of PEDCs on human health problems.

## Figures and Tables

**Table 1:** Binding free energy and its terms (kcal/mol) estimated via the MM-PBSA method.

System	hERRγ-4sec	hERRγ-2nap	hERRγ-BPMe
ΔE_vdW_	−23.95 ± 2.28	−23.54 ± 2.20	−46.47 ± 2.64
ΔE_ele_	−13.02 ± 3.29	−12.54 ± 6.15	−5.59 ± 4.66
ΔE_(MM)_	−36.97 ± 2.83	−36.08 ± 4.62	−52.06 ± 3.79
ΔG_PB_	17.49 ± 2.98	16.30 ± 3.50	32.29 ± 4.45
ΔG_SA_	−1.67 ± 0.08	−1.56 ± 0.09	−3.73 ± 0.12
ΔGsol	15.82 ± 2.11	14.74 ± 2.48	28.56 ± 3.15
ΔG_polar_	4.47 ± 3.14	3.76 ± 5.00	26.70 ± 4.56
ΔG_nonpol_	−25.62 ± 1.61	−25.10 ± 1.55	−50.20 ± 1.87
ΔH	−21.15 ± 3.29	−21.33 ± 3.82	−23.49 ± 3.79
−TΔS	15.21 ± 3.04	15.66 ± 3.10	20.52 ± 3.91
ΔG_bind_	−5.94 ± 3.17	−5.67 ± 3.31	−2.97 ± 3.85
ΔG_exp_	−9.49	−8.98	

Notes: Data are expressed as value ± standard deviation. ΔH = ΔG_polar_ + ΔG_nonpol_ = ΔE_ele_ + ΔG_PB_ + ΔE_vdW_ + ΔG_SA_. Experimental energy (ΔG_exp_) was calculated from inhibition constants (K_i_) using ΔG_exp_ = RTlnK_i_.

**Table 2:** Binding free energy and its terms (kcal/mol) computed using the SIE method.

System	hERRγ-4sec	hERRγ-2nap	hERRγ-BPMe
ΔE_vdW_	−27.63 ± 2.17	−25.93 ± 2.26	−28.37 ± 2.83
ΔE_Coul_	−7.21 ± 1.42	−8.16 ± 2.74	−2.56 ± 2.37
ΔG_cav_	−5.49 ± 0.24	−6.11 ± 0.27	−5.68 ± 0.61
ΔG^R^	4.17 ± 0.74	5.34 ± 0.99	9.96 ± 1.69
ΔG_nonpol_	−33.12 ± 1.13	−32.04 ± 1.61	−34.05 ± 2.06
ΔG_pol_	−3.04 ± 1.54	−2.82 ± 1.94	7.4 ± 2.05
ΔG_bind_	−6.68 ± 0.21	−6.54 ± 0.24	−5.68 ± 0.37
ΔG_exp_	−9.49	−8.98	

Notes: Data are expressed as value ± standard deviation. ΔG_pol_ = ΔG^R^ + ΔE_Coul_, ΔG_nonpol_ = ΔG_cav_ + ΔE_vdw_. Experimental energy (ΔG_exp_) was calculated from inhibition constants (K_i_) using ΔG_exp_ = RTlnK_i_.

## Data Availability

The data used to support the findings of this study are included in the article.

## Supplementary Materials

The following supporting information can be downloaded at: https://www.mdpi.com/article/10.3390/molecules29143256/s1, Tables S1–S3. Different energy components of important residues. Table S4. Important hydrogen bonds between PEDCs and hERRγ. Figure S1. The structural domains of ERRs. Figure S2. Time series of RMSDs. Figure S3. hERRγ secondary structure evolution. Figures S4–S5. Change in enthalpies and entropies and cumulative mean values. Figure S6. RMSF values of Cα atoms. Figure S7. Cross-correlation matrices. Figure S8. PC1 mode for hERRγ. Figure S9. Representative conformation superimposition. Figure S10. Structural superimposition.
