# Supplementary material for: Insight into the Binding Interaction between PEDCs and hERRγ Utilizing Molecular Docking and Molecular Dynamics Simulations
Source: Molecules. 2024 Jul 10;29(14):3256. doi: 10.3390/molecules29143256 (PMC11278984; doi:10.3390/molecules29143256)
Supplement: Supplementary file 1 [file molecules-29-03256-s001.zip › molecules-3059599-supplementary.pdf]

Supporting Information

Insight into the binding interaction between PEDCs and hERRγ utilizing Molecular docking and Molecular dynamics simulations

Author

Fanqiang Bu [1,2], Lin Chen [1,*], Ying Sun [1], Bing Zhao [1,3] and Ruige Wang [1,*]

[1] College of Chemistry and Chemical Engineering, Qiqihar University, Qiqihar 161006, China
[2] State Key Laboratory of Organic-Inorganic Composites, Beijing Laboratory of Biomedical Materials, Beijing University of Chemical Technology, Beijing 100029, China
[3] Heilongjiang Provincial Key Laboratory of Surface Active Agent and Auxiliary, Qiqihar University, Qiqihar 161006, China
* Correspondence: chenlin201308@163.com (L.C.); wangrg716@163.com (R.W.)

Number of pages: 12
Number of figures: 9
Number of tables: 4

Table of contents

Table S1. Each energy term (kcal/mol) contributed by significant residues in hERRγ-4-sec-butylphenol complex……………………………………………………….S2
Table S2. Each energy term (kcal/mol) contributed by significant residues in the hERRγ-tetrahydro-2-napthol complex…………....………………………………...…..S3
Table S3. Each energy term (kcal/mol) contributed by significant residues in hERRγ-BP(2,2)(Me) complex.……………....…………....…………....………….………...…S4
Table S4. Hydrogen binds forming by PEDCs with hERRγ…………………….……….…S5
Figure S1. The structure domain of ERRs………………………….……………………..S5
Figure S2. Time series of RMSD of heavy atoms during three repeated simulations……....S6
Figure S3. Secondary structure evolution of hERRγ over time…………………………..S7
Figure S4. Fluctuations and accumulated mean values of enthalpies………….....................S8
Figure S5. Fluctuations and accumulated mean values of entropies………........................S9
Figure S6. RMSF values of Cα atoms……………………………………….……..S10
Figure S7. Cross-correlation matrices for Cα atoms……………………………….……S11
Figure S8. Procupine representation of PC1 mode…………….………………….…….S12
Figure S9. Representative conformation superimposition for different complexes…….…S12
Figure S10. The structure superimposition…………….……………………………..S12

Table S1. Each energy term (kcal/mol) contributed by significant residues in hERRγ-4-sec-butylphenol complex.

Residue	S_{vdw}	B_{vdw}	T_{vdw}	S_{ele}	B_{ele}	T_{ele}	S_{pb}	B_{pb}	T_{pb}	S_{np}	B_{np}	T_{np}	T_{GBTOT}
Leu268	-0.769	-0.378	-1.146	-0.010	0.147	0.137	0.131	0.046	0.177	-0.086	-0.005	-0.091	-0.923
Leu 271	-0.568	-0.339	-1.146	-0.151	-0.131	-0.282	-0.083	-0.187	-0.270	-0.033	-0.000	-0.033	-1.493
Ala 272	-0.414	-0.669	-1.083	0.018	-0.166	-0.148	0.045	-0.036	0.009	-0.098	-0.022	-0.120	-1.342
Glu 275	0.413	-0.078	0.335	-8.041	-0.076	-8.117	5.793	-0.094	5.699	-0.036	0.000	-0.036	-2.119
Met 306	-0.543	-0.069	-0.611	-0.058	-0.087	-0.145	0.030	0.063	0.093	-0.062	0.000	-0.062	-0.726
Leu 309	-0.904	-0.457	-1.361	0.035	-0.262	-0.227	0.008	0.640	0.648	-0.092	-0.009	-0.101	-1.041
Ile 310	-0.496	-0.286	-0.782	0.059	-0.072	-0.013	-0.093	0.044	-0.049	-0.062	-0.003	-0.065	-0.908
Val 313	-0.778	-0.137	-0.915	-0.036	0.050	0.014	-0.114	-0.141	-0.255	-0.085	-0.000	-0.085	-1.240
Tyr 326	-1.022	-0.238	-1.259	-0.305	-0.010	-0.315	0.617	0.073	0.690	-0.117	-0.002	-0.119	-1.003
Phe 435	-0.860	-0.028	-0.888	0.000	0.019	0.020	0.187	-0.062	0.126	-0.133	0.000	-0.133	-0.876

Table S2. Each energy term (kcal/mol) contributed by significant residues in the hERRγ-tetrahydro-2-napthol complex.

Residue	S_{vdw}	B_{vdw}	T_{vdw}	S_{ele}	B_{ele}	T_{ele}	S_{pb}	B_{pb}	T_{pb}	S_{np}	B_{np}	T_{np}	T_{GBTOT}
Leu268	-0.687	-0.294	-0.981	0.025	-0.580	-0.555	-0.018	0.399	0.381	-0.074	-0.010	-0.084	-1.239
Leu271	-0.549	-0.248	-0.797	-0.061	0.033	-0.028	-0.059	-0.268	-0.327	-0.042	-0.000	-0.042	-1.194
Ala272	-0.285	-0.535	-0.820	0.024	-0.011	0.013	0.038	-0.206	-0.168	-0.078	-0.018	-0.096	-1.070
Glu275	-0.086	-0.056	-0.142	-2.761	-0.056	-2.817	2.285	-0.147	2.138	-0.041	0.000	-0.041	-0.862
Met306	-0.439	-0.124	-0.563	-0.033	-0.069	-0.102	0.092	0.046	0.138	-0.050	-0.000	-0.050	-0.578
Leu309	-0.984	-0.530	-1.514	-0.046	-0.074	-0.120	-0.013	0.356	0.343	-0.130	-0.012	-0.142	-1.434
Ile310	-0.465	-0.366	-0.831	0.072	0.016	0.088	-0.046	-0.011	-0.057	-0.049	-0.003	-0.052	-0.852
Val313	-0.609	-0.190	-0.798	0.009	0.080	0.089	-0.271	-0.175	-0.447	-0.085	-0.001	-0.086	-1.243
Tyr326	-1.220	-0.251	-1.471	-0.237	-0.165	-0.402	0.413	0.157	0.570	-0.147	-0.010	-0.157	-1.462
Phe435	-0.613	-0.021	-0.634	-0.002	0.011	0.009	0.161	-0.041	0.120	-0.117	-0.000	-0.117	-0.621

Table S3. Each energy term (kcal/mol) contributed by significant residues in the hERRγ-BP(2,2)(Me) complex.

Residue	S_{vdw}	B_{vdw}	T_{vdw}	S_{ele}	B_{ele}	T_{ele}	S_{pb}	B_{pb}	T_{pb}	S_{np}	B_{np}	T_{np}	T_{GBTOT}
Leu 268	-1.637	-0.238	-1.875	-0.070	-0.032	-0.102	-0.014	-0.015	-0.029	-0.219	-0.004	-0.223	-2.229
Leu 271	-0.548	-0.068	-0.616	-0.022	-0.028	-0.050	-0.054	-0.176	-0.231	-0.054	-0.000	-0.054	-0.950
Met 306	-0.296	-0.069	-0.365	-0.206	0.045	-0.161	0.088	-0.094	-0.006	-0.030	-0.000	-0.030	-0.562
Leu 309	-0.817	-0.224	-1.041	-0.082	0.112	0.031	-0.034	-0.143	-0.177	-0.090	-0.000	-0.090	-1.277
Ile 310	-0.432	-0.234	-0.666	-0.057	0.093	0.036	-0.006	0.008	0.002	-0.032	-0.001	-0.033	-0.661
Val313	-1.274	-0.180	-1.454	0.077	-0.234	-0.157	-0.381	0.016	-0.365	-0.189	-0.000	-0.189	-2.165
Leu 324	-0.985	-0.645	-1.629	-0.214	-0.860	-1.074	0.057	1.115	1.173	-0.089	-0.080	-0.168	-1.699
Tyr 326	-1.130	-0.330	-1.460	-0.035	0.032	-0.003	0.343	-0.093	0.250	-0.115	-0.010	-0.125	-1.337
Tyr 330	-1.201	-0.105	-1.306	-0.462	-0.092	-0.554	0.946	0.113	1.059	-0.165	-0.004	-0.169	-0.970
Ile 331	-0.591	-0.150	-0.741	-0.024	-0.169	-0.192	-0.021	0.112	0.091	-0.122	-0.004	-0.126	-0.969
Met 332	-1.094	-0.394	-1.488	-0.172	0.161	-0.011	0.322	0.350	0.673	-0.127	-0.052	-0.180	-1.006
Leu 342	-0.746	-0.495	-1.241	0.076	-0.085	-0.008	-0.051	0.127	0.076	-0.082	-0.010	-0.092	-1.266
Leu 345	-0.825	-0.351	-1.176	0.054	0.180	0.234	0.018	-0.080	-0.062	-0.092	-0.001	-0.092	-1.096
Asn 346	-1.313	-0.574	-1.887	-0.787	-0.020	-0.807	1.033	-0.205	0.828	-0.240	-0.018	-0.258	-2.124
Ile 349	-0.708	-0.061	-0.769	-0.098	0.015	-0.083	0.060	-0.096	-0.036	-0.085	0.000	-0.085	-0.973

Table S4. Hydrogen bonds between PEDCs and significant residues.

Donor	Acceptor	4-sec-Butylphenol-hERRγ	Tetrahydro-2-napthol-hERRγ	BP(2,2)(Me)-hERRγ
Glu275@OE1	SEC@OAC/H12	86.93		
SEC@OAC	Met@NH1/H12	18.93		
293@O	2NAP@NZ/H		28.56	
297@OE1	2NAP@NZ/H		25.76	
BPME@OXT	415@NH2/H			36.73
BPME@O	415@NH1/H			27.13

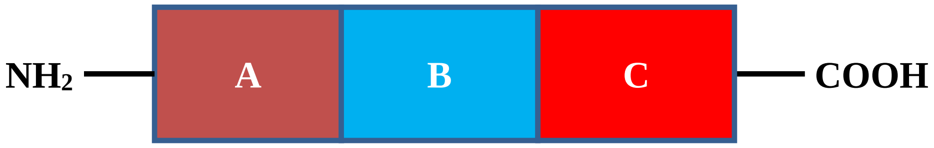

Figure S1. The structural domains of ERR

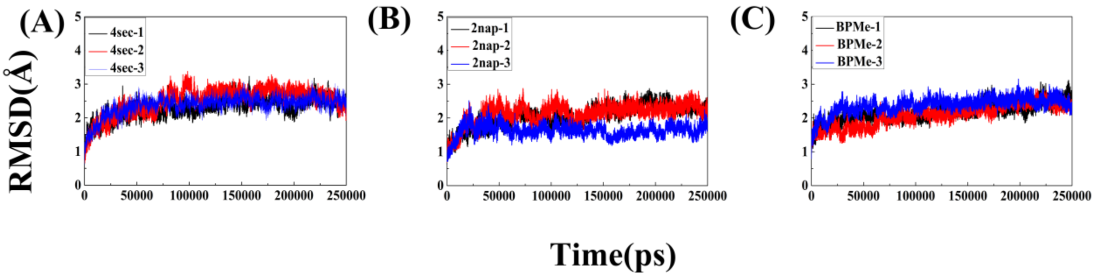

Figure S2. Time series of RMSD of heavy atoms during three repeated simulations for different complexes: (A) hERRγ-4-sec-butylphenol; (B) hERRγ-tetrahydro-2-napthol; (C) hERRγ-BP(2,2)(Me).

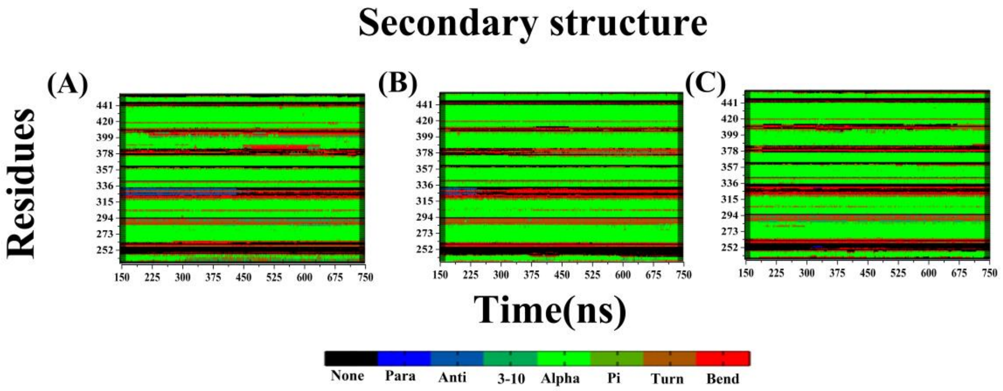

Figure S3. Secondary structure evolution of HERRγ over time: (A) hERRγ-4-sec-butylphenol complex; (B) hERRγ-tetrahydro-2-napthol complex; (C) hERRγ-BP(2,2)(Me) complex.

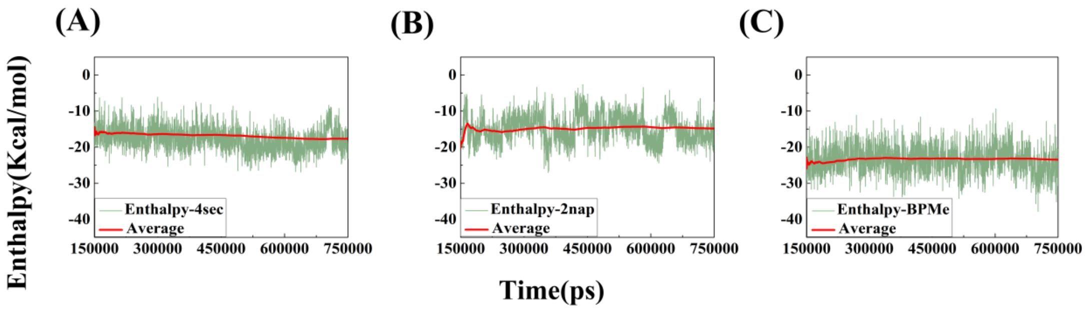

Figure S4. Fluctuations and accumulated mean values of enthalpies: (A) hERRγ-4-sec-butylphenol complex; (B) hERRγ-tetrahydro-2-napthol complex; (C) hERRγ-BP(2,2)(Me) complex.

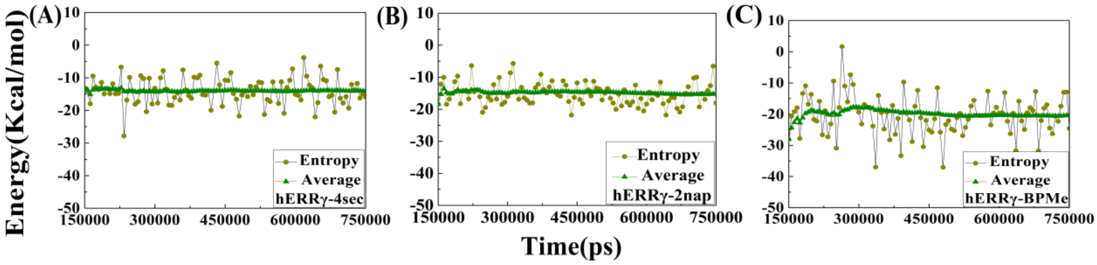

Figure S5. Fluctuations and accumulated mean values of entropies: (A) hERRγ-4-sec-butylphenol complex; (B) hERRγ-tetrahydro-2-napthol complex; (C) hERRγ-BP(2,2)(Me) complex.

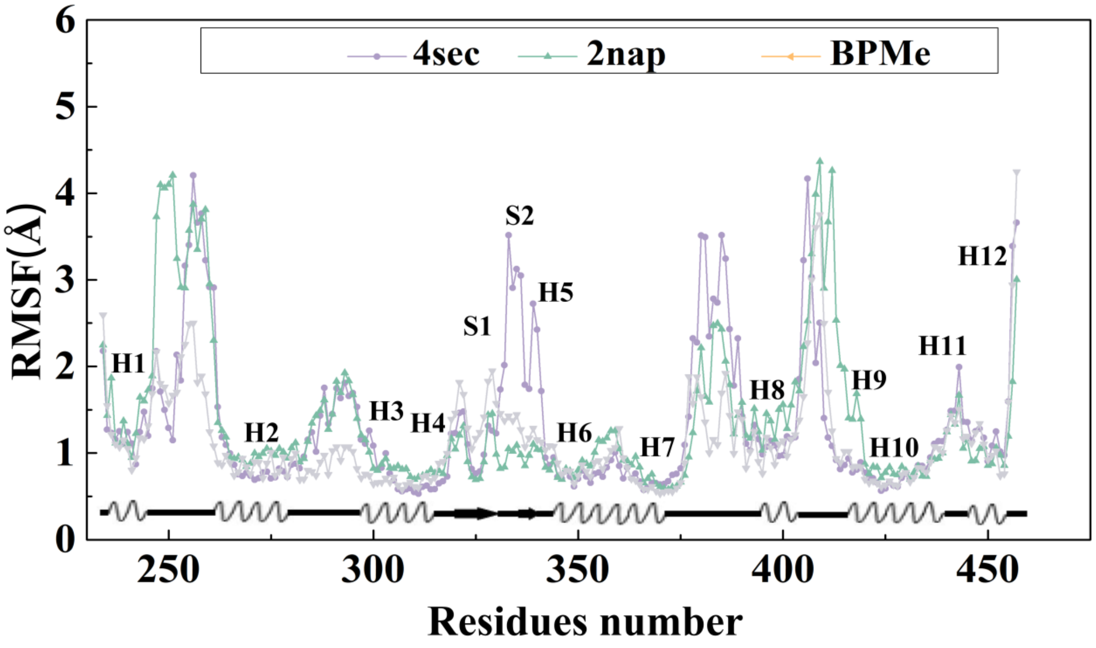

Figure S6. RMSF values of Cα atoms relative to initial structure in hERRγ-PEDCs complexes.

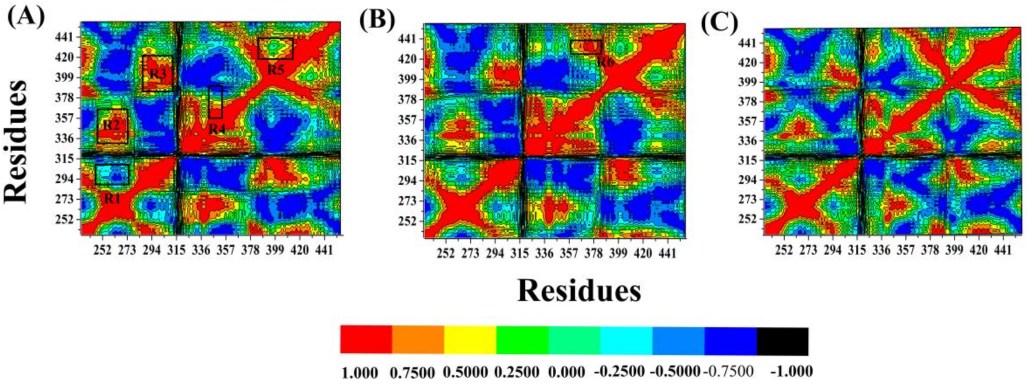

Figure S7. Cross-correlation matrices for Cα atoms: (A) hERRγ-4-sec-butylphenol complex; (B) hERRγ-tetrahydro-2-napthol complex; (C) hERRγ-BP(2,2)(Me) complex.

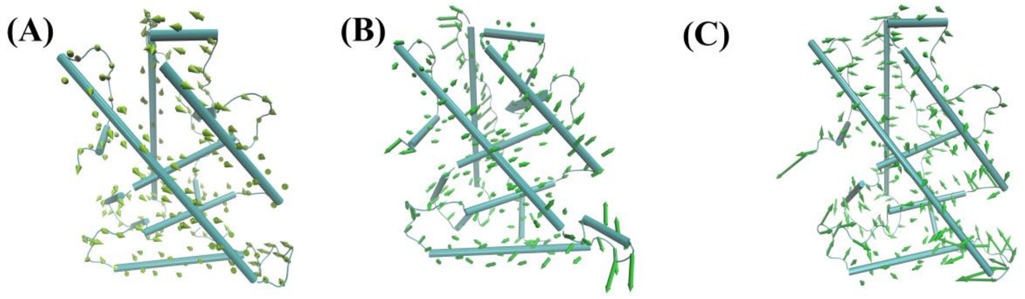

Figure S8. Procupine representation of PC1 mode: (A) hERRγ-4-sec-butylphenol complex; (B) hERRγ-tetrahydro-2-napthol complex; (C) hERRγ-BP(2,2)(Me) complex.

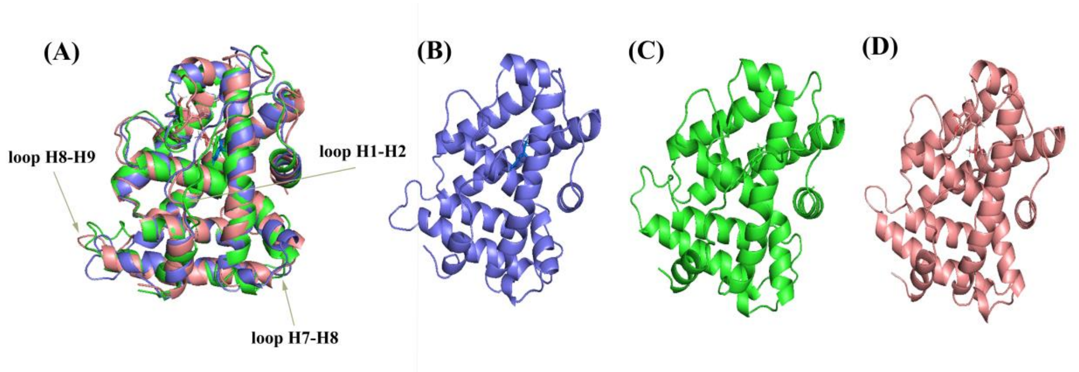

Figure S9. Representative conformation Superimposition for different complexes (green indicates hERRγ-4-sec-butylphenol, magenta indicates hERRγ-tetrahydro-2-napthol, oranges indicates hERRγ-BP(2,2)(Me) complex.

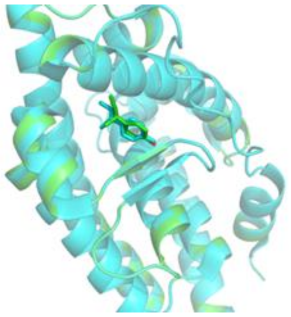

Figure S10. The structure superimposition of 4-sec-butylphenol- hERRγ between docking result (green) and crystal structure (Cyan)
